# Comparative Study of Antimicrobial Activity of AgBr and Ag Nanoparticles (NPs)

**DOI:** 10.1371/journal.pone.0119202

**Published:** 2015-03-17

**Authors:** Petr Suchomel, Libor Kvitek, Ales Panacek, Robert Prucek, Jan Hrbac, Renata Vecerova, Radek Zboril

**Affiliations:** 1 Regional Centre of Advanced Technologies and Materials, Faculty of Science, Palacky University Olomouc, Czech Republic; 2 Department of Physical Chemistry, Faculty of Science, Palacky University Olomouc, Czech Republic; 3 Department of Microbiology, Faculty of Medicine and Dentistry, Palacky University Olomouc, Czech Republic

## Abstract

The diverse mechanism of antimicrobial activity of Ag and AgBr nanoparticles against gram-positive and gram-negative bacteria and also against several strains of candida was explored in this study. The AgBr nanoparticles (NPs) were prepared by simple precipitation of silver nitrate by potassium bromide in the presence of stabilizing polymers. The used polymers (PEG, PVP, PVA, and HEC) influence significantly the size of the prepared AgBr NPs dependently on the mode of interaction of polymer with Ag^+^ ions. Small NPs (diameter of about 60–70 nm) were formed in the presence of the polymer with low interaction as are PEG and HEC, the polymers which interact with Ag^+^ strongly produce nearly two times bigger NPs (120–130 nm). The prepared AgBr NPs were transformed to Ag NPs by the reduction using NaBH_4_. The sizes of the produced Ag NPs followed the same trends – the smallest NPs were produced in the presence of PEG and HEC polymers. Prepared AgBr and Ag NPs dispersions were tested for their biological activity. The obtained results of antimicrobial activity of AgBr and Ag NPs are discussed in terms of possible mechanism of the action of these NPs against tested microbial strains. The AgBr NPs are more effective against gram-negative bacteria and tested yeast strains while Ag NPs show the best antibacterial action against gram-positive bacteria strains.

## Introduction

Silver nanoparticles (Ag NPs) and nanoparticles of insoluble silver compounds such as Ag_2_O, AgOH or AgX (X = Cl, Br or I) became one of the most discussed branches of nanoscience in the several last decades [1]. Ag NPs attract great attention in the scientific research because of their unique physical, chemical, optical and biological properties which predetermine them for use in various chemical, medical and industrial applications [2]. Nowadays, diverse biological properties, namely antifungal, antiviral and particularly antibacterial activities together with cytotoxicity of silver NPs are intensely studied because of expected inability of bacteria to develop resistance against their antibacterial action. According to this, silver based nanomaterials find their use in medicine (catheters, implants, prostheses) [3–7], and are used to improve commercial products (textiles, deodorants) [8–10].

The antibacterial effect of silver based nanomaterials, such as metallic silver nanoparticles as well as nanoparticles of silver compounds, was reported by many researchers [11–12], but unfortunately, the reported results were not compared with each other. It was revealed that antibacterial effect is not specific at a single level but consists of several modes of action. It was shown, that silver based nanomaterials can disrupt bacterial metabolic processes [13–14], interact with DNA [15], increase the cytoplasmic membrane permeability [16–17] etc. Due to this multi-level mode of action, silver based nanomaterials reveal extraordinary activity not only against sensitive bacterial strains but also against highly resistant bacterial strains [18–19]. On the other hand it was observed resistance to ionic silver [20]. Despite the fact, that resistance to ionic silver originating from the ability of bacteria to reduce Ag^+^ to less toxic oxidation state or from active efflux of Ag^+^ from the cell was reported [21], no data proving resistance to metallic Ag nanoparticles were published. Naturally, the antibacterial effect of silver and silver based nanoparticles is dependent on particle morphology and surface characteristics [22–24]. Hence, it is necessary to find nanoparticles by the means of size, shape and surface modification appropriate for use in the biological applications.

Silver NPs can be prepared many different ways but wet chemical reduction methods unambiguously predominate. Silver NPs can be prepared by the reduction of either, soluble and insoluble silver compounds [25]. Typical synthetic method is based on the reduction of silver nitrate by sodium borohydride which is one of the strongest reducing agents and therefore very small Ag NPs are produced [26]. However, a great number of milder reducing agents can be used for the production of silver NPs with various sizes, size distributions, shapes and morphologies [27–30]. A noteworthy example is e.g. the utilization of reducing saccharides which are week reducing agents allowing for the preparation of bigger Ag NPs with sizes in the range of several tens of nm [31–32]. In addition to widespread use of soluble silver salts, insoluble Ag(I) compounds can also be used as starting materials for the silver nanoparticles’ preparation. Commonly, insoluble Ag(I) compounds are prepared in the form of nanoparticles by precipitation reactions of silver salt and appropriate reactant containing suitable counterion (e.g. hydroxide, halide, sulfide etc.) The most studied insoluble Ag(I) compounds are Ag_2_O [33–34], Ag_2_S [35–37], AgOH [38] or AgX (where X = Cl, Br or I) [39–41]. The nanoparticles of silver halides can be prepared in the variety of morphological forms. Typically, the AgCl NPs can be prepared in the form of cubic nanoparticles with sizes between 120 and 250 nm [42] or nanowires that are composed from nanoparticles with diameters of tens of nanometers [43]. AgBr NPs can be prepared in the form of spheres with sizes of units of nm [44], spherical nanoparticles with sizes from 70 up to 140 nm [45], porous spherical structures with average dimensions of about 150–200 nm and pore size of about 5–10 nm [46] or bipyramids and polyhedrons [47]. Finally, AgI NPs can be prepared in the form of plate-like nanoparticles with polygonal faces of diameter equal to 300 nm and the thickness of 50 nm [48] as well as also in the form of spherical nanoparticles with diameter of several tens of nm [49–50] or hundreds nm [51]. The primarily prepared silver insoluble compound nanoparticles can be converted by the reduction to the Ag NPs. Besides chemical reduction method, electrochemical reduction or photoreduction by UV irradiation (especially in the case of silver halides) can also be used for this purpose [52–54]. In the case of chemical reduction a variety of reducing agents can be used similarly to the case of soluble silver compounds’ reduction. Naturally, the use of different reducing agents leads to the formation of Ag NPs with various sizes and shapes [55–56]. Despite the fact that Ag NPs preparation methods based on the reduction of insoluble silver salts are not as widely examined as is the reduction of soluble silver salts, insoluble silver salt reduction methods could pose some advantages such as simple preparation of thin films or composites because of better affinity of silver compounds to the carrier compared to metallic silver [57–59]. Also, preparation of stable nanoparticles with very small diameters is feasible using this method [60].

Therefore, preparation of AgBr nano and submicro particles, their subsequent reduction to the silver NPs and the comparison of antibacterial activities of them are the main objective of this work. AgBr was precipitated in the presence of various polymer stabilizers in order to prepare suitable and stable particles for their subsequent transformation to Ag NPs by the reduction process. Antimicrobial activities of both AgBr particles and silver NPs were compared.

## Materials and Methods

### Chemical and biological material

Silver nitrate (Sigma-Aldrich, p.a.) and potassium bromide (Lachema, p.a.) were used for the synthesis of AgBr particles. Polyethylene glycol (PEG, M.W. 10 000, Sigma-Aldrich, p.a.), polyvinyl pyrrolidone 360 (PVP, M.W. 360 000, Sigma-Aldrich, p.a.), polyvinyl alcohol (PVA, M.W. 85 000–146 000, Sigma-Aldrich, 98–99% hydrolyzed) and 2-hydroxyethyl cellulose (HEC, M.W. 90 000, Sigma-Aldrich) were used as stabilizing substances in the process of AgBr particles’ preparation. Prepared AgBr particles were reduced by using of sodium borohydride (Sigma-Aldrich, p.a.). All chemicals were used without additional purification. Deionized water (18 MΩ·cm, Millipore) was used to prepare all solutions.

For the purpose of antibacterial and antifungal assays Mueller-Hinton broth (Difco Becton Dickinson) was used as a cultivation medium. The following reference strains (labeling according to Czech Collection of Microorganisms, Czech Republic) were used: *Enterococcus faecalis* CCM 4224, *Staphylococcus aureus* CCM 3953, *Escherichia coli* CCM 3954, *Pseudomonas aeruginosa* CCM 3955. The strains isolated from the blood of patients hospitalized at the University Hospital Olomouc (Czech Republic): *Pseudomonas aeruginosa*, *Staphylococcus epidermidis*, *Staphylococcus aureus* (MRSA), *Enterococcus faecium* (VRE), *Klebsiella pneumoniae* (ESBL), *Candida albicans* I, *Candida albicans* II, *Candida tropicalis* and *Candida parapsilosis* were also used.

### Characterization techniques

The prepared silver bromides as well as metallic silver nanoparticles were characterized by dynamic light scattering (Zeta Plus, Brookhaven Instr. Co., USA) and UV-Vis spectroscopy (Specord S600, Analytic Jena AG, Germany) techniques. Additionally, AgBr NPs were characterized by atomic force microscopy (NTEGRA Aura, NT-MDT, Russia), and silver nanoparticles were characterized by transmission electron microscopy (JEM 2010, Jeol Ltd., Japan).

### Synthesis of AgBr nanoparticles

The colloidal dispersions of silver bromide nanoparticles were prepared by rapid injection of potassium bromide into the solution of silver nitrate under vigorous stirring at final concentrations of 2·10^-3^ mol/L (KBr) and 1·10^-3^ mol/L (AgNO_3_) respectively in the reaction system. For the purpose of size modification and stabilization of AgBr NPs formed in the reaction system three synthetic (PVA, PEG and PVP) and one natural polymer (polysaccharide HEC) were tested as stabilizers. Stabilizers were added into KBr solution prior to the precipitation at the final concentration in resulting AgBr dispersion equal to 0.1% (w/w). Five minutes after precipitation, the sizes and size distributions of the prepared AgBr NPs were determined by dynamic light scattering (DLS) technique. At the same time the samples for the determination of size and morphology of prepared silver bromide nanoparticles by AFM were collected. For the purpose of AFM measurements, a drop of AgBr dispersion was spread on foil and allowed to dry at 50°C in the dark. The attempt at characterizing the prepared AgBr particles by transmission electron microscopy (TEM) was unsuccessful due to rapid reduction of AgBr particles in the electron beam of the microscope.

### Synthesis of Ag NPs by reduction of AgBr nanoparticles

For the synthesis of Ag NPs, freshly prepared AgBr dispersions were reduced by using NaBH_4_. AgBr NPs were reduced by rapid injection of 5 ml of 6·10^-2^ mol/L sodium borohydride into the 25 ml of AgBr dispersion under vigorous stirring. Fresh solutions of sodium borohydride were applied for the reduction process which was performed at the laboratory temperature. The reduction process was completed after 20 minutes when color of the reaction system changed to light or dark yellow (depending on used polymer), indicating the presence of silver nanoparticles. Final concentration of sodium borohydride was 1·10^-2^ mol/L. The prepared silver nanoparticles were characterized by DLS, UV-Vis spectroscopy and TEM methods. Additionally, kinetic curves of silver bromide reduction were recorded by using of the UV-Vis spectroscopy.

### Antibacterial and antifungal assay

Antibacterial and antifungal efficacies of the prepared AgBr and Ag NPs were tested using the standard dilution method which enables to determine the minimum inhibitory concentrations (MICs) of the tested samples necessary to inhibit the growth of the bacterial strains and yeasts. For the purpose of antimicrobial testing, aqueous dispersions of AgBr and Ag NPs at the silver concentration equal to 108 mg/L for AgBr colloid and 89.6 mg/L for Ag dispersions were used. The testing was carried out in microtitration plates where the tested samples were diluted by the culture medium (Mueller Hinton Broth, Difco, France) in a geometric progression from 2 to 128 times. Culture medium was inoculated with the tested bacteria or yeast at a concentration of 10^5^ to 10^6^ CFU/mL. After 24-h incubation at 37°C, the MICs of AgBr and Ag nanoparticles were read as the lowest concentrations of the tested substance inhibiting the visible growth of microorganisms.

## Results and Discussion

### Synthesis of AgBr nanoparticles

**Fig 1:**
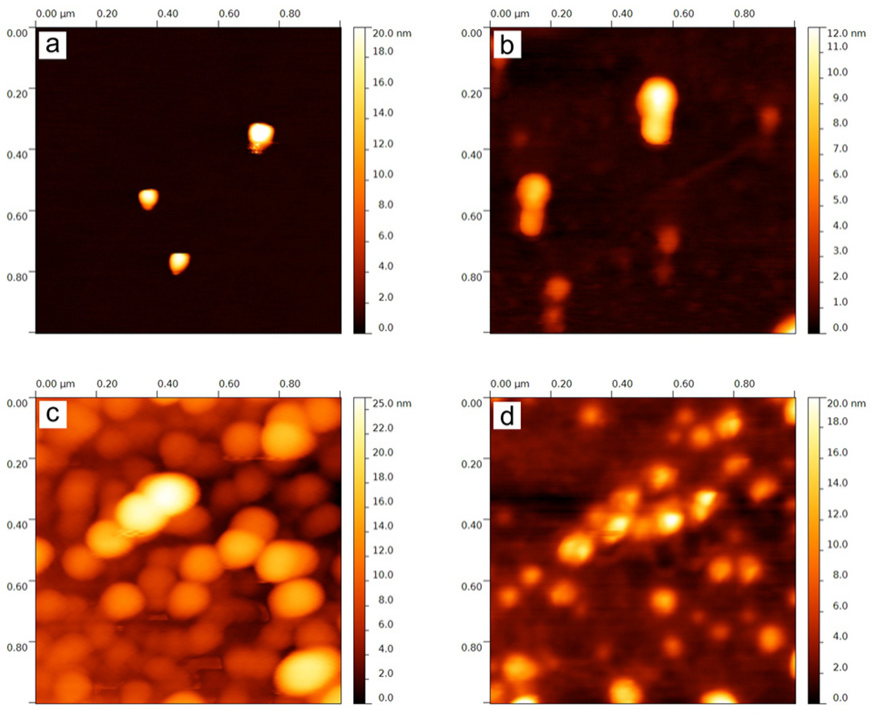
AFM images of AgBr nanoparticles prepared in the presence of a) PEG, b) PVP, c) PVA and d) HEC.

Silver bromide nanoparticles were prepared simply by mixing the reaction components at desired ratio in the presence of four different polymers which affect the morphology and size of the prepared AgBr NPs. Due to the problem with reduction of AgBr NPs by electron beam of the electron microscope, the AFM measurements were utilized as primary characterization technique. Images from AFM (Fig. 1) show that prepared silver bromide nanoparticles have rotational ellipsoidal shape with average height from 11 nm up to 18 nm and average diameter from 60 nm up to 160 nm depending of the used polymer additive (Table 1).

**Table 1:** Particle sizes of AgBr nanoparticles prepared in the presence of various polymers obtained by AFM and DLS techniques.

Polymer	AFM (nm)		DLS (nm)
**PEG**	height	18	105
	diameter	64	
**PVP**	height	10	50
	diameter	120	
**PVA**	height	11	70
	diameter	30	
**HEC**	height	12	100
	diameter	70	

The used polymers can be divided into two groups based on the level of interaction with Ag^+^ ions in the solution. The level of interaction is primarily dependent on the chemical composition of each polymer and on the polymer structure. The first group contains PVP and PVA polymers which functional groups are located outside the polymer chain. These are—OH group in the case of PVA and = O group in the case of PVP. Additionally, PVP contains atoms of nitrogen in the molecule and therefore it can interact with Ag^+^ ions as a Lewis base. The Ag^+^ ions bind with these functional groups of PVA and PVP respectively to form complexes in the reaction system [61–65]. The complexation of Ag^+^ ions is slowing down the formation of new phase nuclei during the precipitation leading to a smaller amount of nuclei formed in each reaction system. This effect results in the formation of larger AgBr particles in the presence of PVP and PVA polymers [66]. Even though PEG and HEC also contain oxygen heteroatoms in their molecules, these polymers do not interact with silver as strongly as PVP or PVA. For these polymers, the formation of complexes with Ag^+^ ions has not been described in the literature. This is because of oxygen atoms are incorporated into polymer chain and therefore they cannot participate on the coordination bond with Ag^+^ ions. Weak interaction of PEG and HEC with silver ions results in the formation of more nuclei and leads to the growth of smaller AgBr NPs compared to those prepared in the presence of PVP and PVA. The same amount of silver in each reaction system must be divided among large number of growing nuclei. This assumption corresponds well with AFM measurements but unfortunately there is a disagreement between the results obtained by AFM and DLS at first sight (Table 1, S1 Fig.). This discrepancy can be explained by the affinity of functional groups of used polymers to silver. PVP and PVA are much more strongly adsorbed onto the surfaces of AgBr NPs than PEG and HEC. Therefore, AgBr NPs readily aggregate in the presence of PEG and HEC which are not strong stabilizing agents and the aggregates negatively affect the results of DLS measurements.

### Synthesis of Ag NPs by reduction of AgBr nanoparticles

**Fig 2:**
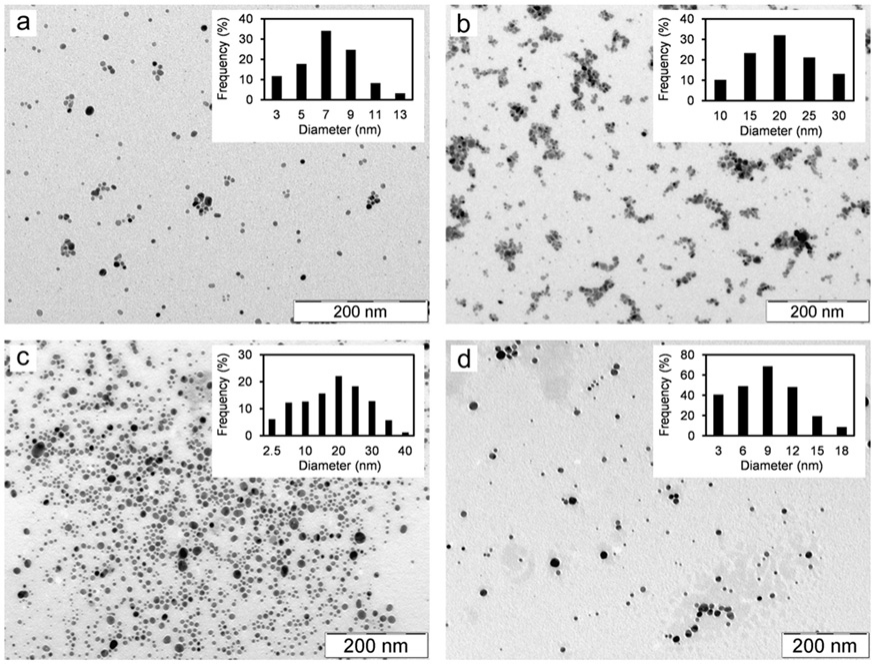
TEM images and distribution diagrams of Ag NPs prepared in the presence of a) PEG, b) PVP, c) PVA and d) HEC.

Silver nanoparticles were prepared by the reduction of previously prepared AgBr by NaBH_4_ and were characterized by DLS, TEM and UV-Vis spectroscopy. The results obtained from DLS measurements (Table 2, S2 Fig.) indicate the presence of silver NPs with various sizes from 8 up to 40 nm depending on the used polymer. The effective diameters of silver nanoparticles measured by DLS were verified by transmission electron microscopy (Table 2). The obtained TEM images were examined by image analysis which confirmed the formation of silver nanoparticles with average diameter under 10 nm in the presence of PEG and HEC, and with sizes around 20 nm in the case of PVA and PVP additives (Fig. 2).

**Table 2:** Average diameter (nm) / polydispersity of Ag NPs prepared by the reduction of AgBr NPs by the NaBH_4_ in the presence of various polymers obtained by DLS and TEM techniques.

Polymer	DLS	TEM
**PEG**	9 / 0.464	7 / 0.343
**PVP**	21 / 0.195	20 / 0.294
**PVA**	18 / 0.482	18 / 0.517
**HEC**	9 / 0.512	9 / 0.456

**Fig 3:**
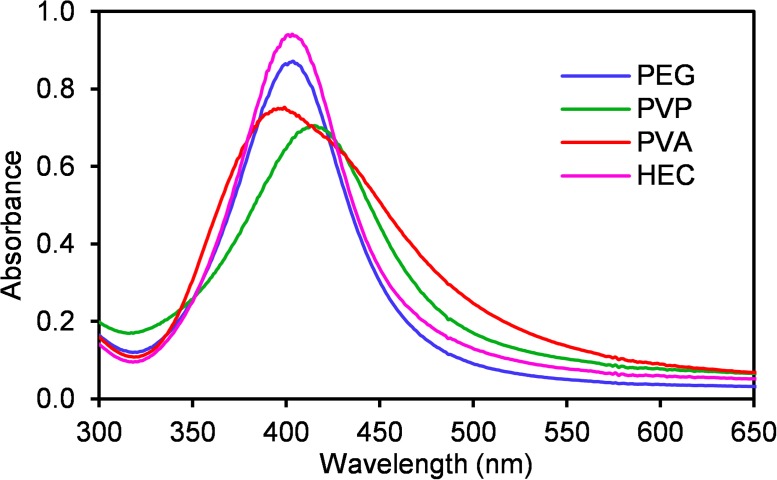
UV-Vis spectra of ten times diluted Ag NPs in aqueous dispersions prepared by the reduction of AgBr NPs by NaBH_4_ in the presence of a) PEG, b) PVP, c) PVA and d) HEC.

**Fig 4:**
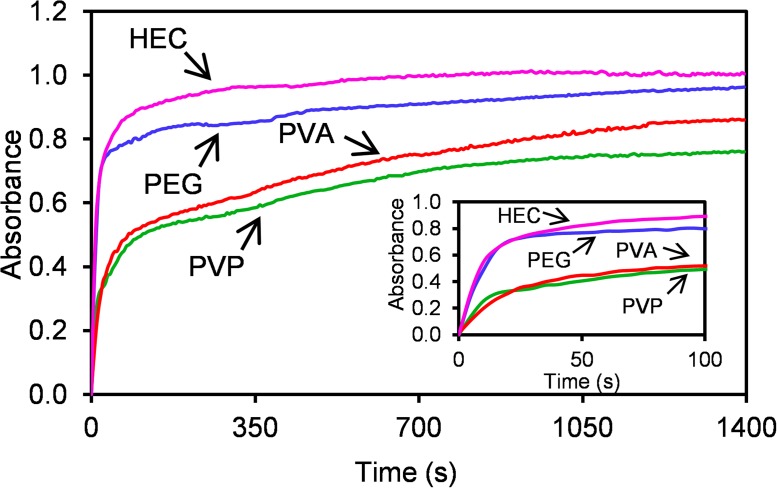
Kinetic curves for AgBr nanoparticles reduction by NaBH_4_ in the presence of studied polymers (λ_max_ represent position of absorption maxima) and zoom of the first 60 seconds (inset).

UV-Vis absorption spectra of all synthesized silver NPs (Fig. 3) confirm the DLS and TEM results. Dispersions of silver NPs stabilized by PEG and HEC reveal strong absorption peak with maximum around the wavelength of 400 nm indicating the presence of small nanoparticles with size of about 10 nm. Silver NPs stabilized by PVP show shift in the absorption peak maximum to the wavelength of 410 nm indicating the presence of nanoparticles bigger than those formed in the systems with PEG and HEC polymers. In the case of PVA, two absorption maxima located at the wavelength below 400 nm and at 420 nm respectively are observed. This fact indicates more polydisperse system, which is also confirmed by TEM images. The first absorption peak can be assigned to very small nanoparticles with diameter less than 10 nm. The second peak can be attributed to the bigger nanoparticles with sizes up to 30 nm. In order to determine of the mechanism of added polymers’ influence on the formation of Ag NPs, the kinetics of the AgBr NPs reduction by NaBH_4_ was examined by recording the UV-Vis spectra during the course of reduction process (Fig. 4). Kinetic measurements show different rates of the AgBr NPs reduction depending on the used polymer. On the basis of the overall reduction rate it is possible to divide the reaction systems into two groups which nicely correlate with above discussed mode interaction of Ag^+^ ions and polymers in the reaction system during AgBr precipitation process.

**Fig 5:**
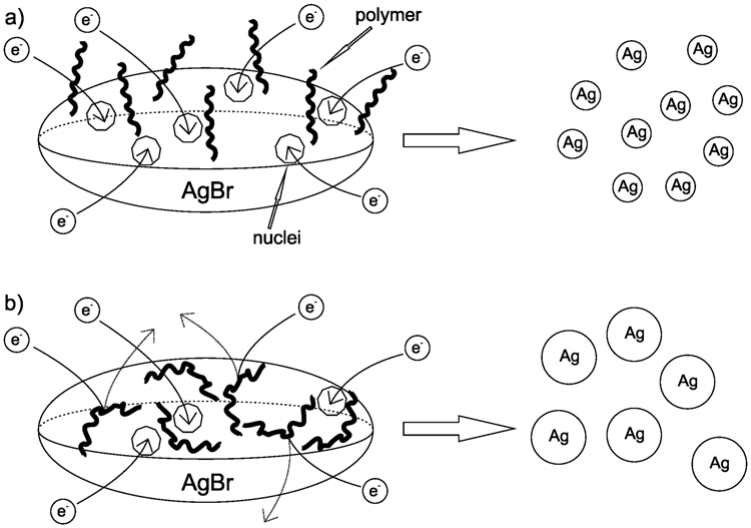
Schematic illustration of nucleation and formation process of Ag NPs from AgBr NPs affected by a) PEG or HEC and b) PVP or PVA.

PVP and PVA polymers exhibit higher affinity to silver which results in blocking of AgBr particle surface during the reduction process. Because of that the reaction takes place only at several locations on particle surface which results in the formation of bigger final silver nanoparticles. On the other hand, faster reduction was observed in the presence of PEG and HEC polymers. These polymers with lower affinity to AgBr surface do not exhibit strong adsorption and consequently higher number of active sites on the particle surface is accessible for the reducing agent. Therefore, reduction of AgBr NPs proceeds much faster on a greater number of active sites and the primary AgBr NPs yield many smaller silver nanoparticles by the reduction process (Fig. 5).

The above discussed impact of the used polymers on AgBr nanoparticles’ reduction was confirmed by performing the electrochemical reduction of Ag^+^ ions in the presence of individual polymers by the technique of cyclic voltammetry on Pt electrode with scan rate of 0.1 V/s. All studied polymers excluding HEC negatively affect the electrochemical reduction of Ag^+^ ions which is reflected by decrease in the reduction currents and by shifting of cathodic peaks toward negative potentials compared to the reduction of pure Ag^+^ ions (S3 Fig.). This effect is mainly caused by the formation of Ag^+^ complexes with added polymers which is connected with observed reduction potential shift. A specific (catalytic) effect on electrochemical process of Ag^+^/Ag was found for HEC polymer. HEC behaves as a weak reducing agent and therefore more complicated cyclic voltammogram was observed in this case especially when the scan rate was increased up to 1 V/s (S4 Fig.). In this case the second reduction peak connected with electrocatalytic effect of HEC is observed and on the reverse scan a sharp increase of anodic current due to direct oxidation of HEC is also observed.

### Antibacterial and antifungal activity of AgBr and Ag nanoparticles

Antibacterial and antifungal activities of the prepared AgBr and Ag NPs were tested on 9 strains of bacteria and 4 strains of candida. Table 3 shows the obtained experimental data in the form of minimum inhibitory concentrations (MIC) that were recalculated on the total concentration (mg/L) of silver in the samples. The presented results represent average values from 3 separate measurements. The obtained values of MIC in the range of 0.8 and 20.2 mg/L demonstrate, that silver bromide and silver nanoparticles prepared by the described method have high antibacterial and antifungal activities that are comparable with previously published values [67–68], and the obtained MIC values remain below cytotoxicity concentrations reported in the literature [69]. Antibacterial and antifungal activities of polymers in appropriate concentrations as blank samples were tested. It was proved that none of the used polymers exhibit any antibacterial or antifungal effects in the used concentrations.

**Table 3:** Minimum inhibitory concentrations (mg/L) of silver bromide nanoparticles, silver nanoparticles and ionic silver against gram-positive (+) and gram-negative (-) bacteria and against yeast pathogens recalculated on total silver concentration prepared in the presence of selected polymers.

	PEG		PVP		PVA		HEC		×
	AgBr	Ag^0^	AgBr	Ag^0^	AgBr	Ag^0^	AgBr	Ag^0^	Ag^+^
*Enterococcus faecalis* CCM 4224 (+)	13.5	5.1	13.5	20.2	13.5	20.2	13.5	5.1	13.5
*Staphylococcus aureus* CCM 3953 (+)	13.5	5.1	13.5	10.1	13.5	5.1	13.5	2.5	13.5
*Escherichia coli* CCM 3954 (-)	3.4	5.1	6.7	5.1	6.7	5.1	6.7	2.5	3.4
*Pseudomonas aeruginosa* CCM 3955 (-)	0.8	2.8	0.8	2.5	0.8	2.5	0.8	2.5	3.4
*Pseudomonas aeruginosa* (-)	0.8	2.5	0.8	2.5	1.7	2.5	1.7	2.5	3.4
*Staphylococcus epidermidis* (+)	1.7	2.5	3.4	2.5	3.4	2.5	3.4	1.3	6.7
*Staphylococcus aureus* (MRSA)(+)	13.5	5.1	13.5	10.1	13.5	5.1	13.5	2.5	13.5
*Enterococcus faecium* (VRE)(+)	13.5	5.1	6.7	10.1	6.7	5.1	6.7	5.1	13.5
*Klebsiella pneumoniae* (ESBL) (-)	3.4	5.1	3.4	5.1	3.4	2.5	3.4	5.1	3.4
**Antifungal effect**									
*Candida albicans* I	1.7	2.5	1.7	2.5	1.7	2.5	1.7	1.3	1.7
*Candida albicans* II	1.7	1.3	3.4	2.5	1.7	2.5	1.7	2.5	3.4
*Candida tropicalis*	3.4	2.5	1.7	2.5	1.7	1.3	1.7	1.3	1.7
*Candida parapsilosis*	1.7	2.5	1.7	2.5	1.7	2.5	1.7	1.3	1.7

Although values of MIC obtained for AgBr and Ag NPs seem nearly the same, there are some interesting trends observed for both colloids. The AgBr NPs are evidently more active against *Pseudomonas aeruginosa* CCM 3955, *Pseudomonas aeruginosa* (wild strain from Olomouc University Hospital), and *Klebsiella pneumonia* (ESBL) (wild strain from Olomouc University Hospital). On the other hand, Ag NPs are more active than AgBr NPs against both tested *Staphylococcus aureus* strains. Additionally, even more interesting differences can be observed in antimicrobial activities of differently modified nanoparticles. The best antimicrobial activity was observed for Ag NPs modified by HEC polymer in which case, ten of the fourteen tested microbial strains were most effectively inhibited by this combination of metal nanoparticles and polymer modifier. It is interesting, that in all these cases the HEC modified Ag NPs have the highest antimicrobial activity not only in comparison with all other combinations of metal nanoparticles and polymer modifiers but also in comparison with ionic silver used as reference nanomaterial.

With respect to the activity of ionic silver it is possible to divide the reported antimicrobial activities of the studied combinations of metal nanoparticles and polymer modifiers into two groups according to the tested microorganisms. While in most cases significantly higher antibacterial activities of the polymer modified metal nanoparticles against bacteria strains are observed compared to antibacterial activities of ionic silver, in the cases of tested yeast pathogens the antimicrobial activities of silver nanoparticles and ionic silver are closely comparable. This observation can be undoubtedly connected to the modes of interactions of the tested materials with the used microbial strains. In the case of bacteria, which are prokaryotic microorganisms, the more effective mechanism of antimicrobial action of silver or silver bromide nanoparticles could be their destructive interaction with the cell wall rather than the penetration of silver ions into the cell and subsequent degradation of their bioactive molecules. This phenomenon was confirmed by TEM observations described in the paper of Mirzajani et al. and other researchers [70–72]. On the other hand, in the case of yeast pathogens, which are eukaryotic organisms, the efficiency of the interaction of metal nanoparticles with cell membrane and penetration of silver ions into cell seem to be nearly the same in the course of antimicrobial action. The more detailed view on the obtained results of the antimicrobial activity of tested antibiotics reveals the highest antimicrobial activity of silver nanoparticles against gram-positive bacteria (*Staphylococcus* and *Enterococcus sp*.). Evidently, the silver nanoparticles strongly interact with the peptidoglycan layer of bacteria wall and disrupt it [70] while interaction of the outer part of bacteria wall with silver ions (coming from both, soluble or insoluble silver salt) is not so devastating. The markedly lower values of MIC for silver nanoparticles in comparison to MIC of Ag^+^ (or AgBr NPs) for most tested gram-positive bacterial strains seems to confirm this mechanism. On the other hand, interaction of silver nanoparticles with outer liposaccharide part of bacterial wall in the case of gram-negative bacteria is not so effective for bacteria growth inhibition in comparison with silver ions penetration through the cell wall inside the bacteria. Thin gram-negative bacteria wall does not constitute such critical barrier for silver ions’ penetration as is the case for substantially thicker gram-positive bacteria cell wall [73]. In the latter case the combination of both above mentioned mechanisms seems to be more effective and therefore AgBr NPs are most efficient from all of the three tested forms of silver. To confirm leaching of Ag+ ions from the presented nanoparticles the concentration of ionic silver in studied systems was determined by electrochemical measurements using ISE electrode (S1 Table). The obtained values ranged around 5·10^-5^ mg/L in the cases of AgBr NPs and around 1·10^-5^ mg/L in the case of Ag NPs. Therefore, the impact of ionic silver is expected to be more significant in the antibacterial activity of AgBr NPs than in the case of Ag NPs. In general, all the tested gram-negative bacteria strains and also yeast pathogens (whose cytoplasmic membrane has similar lipid structure as the outer part of gram-negative bacteria cell wall) show higher sensitivity against antimicrobial action of the tested silver based materials than tested gram-positive bacterial strains. The lower sensitivity of gram-positive bacterial strains against silver based materials is primarily due to the thickness of peptidoglycan layer, which may prevent transport of silver based materials through the bacterial cell wall [71,73]. In the case of gram-negative bacterial strains their sensitivity against silver based materials is very similar to yeast pathogens, therefore the possible influence of differences in composition of the outer part of the cell membrane (existence of outermembrane proteins in gram-negative bacteria) seems to be negligible in the studied case.

## Conclusions

This article describes the preparation of AgBr NPs in the presence of the modifying polymers and their subsequent reduction to metallic Ag NPs using NaBH_4_ as reducing agent. The resulting sizes of the prepared AgBr and Ag NPs significantly depend on used polymer. The main influence on the size of prepared nanoparticles has the process of the complexation of Ag^+^ ions by functional groups of polymers. The formation of complexes between Ag^+^ ions and—OH or = O groups of PVP and PVA respectively leads to the creation of AgBr particles with bigger sizes than those which form in the presence of PEG and HEC. On the other hand, kinetic measurements show blocking of the surface of AgBr nanoparticles by these strongly interacting polymers leading to the slower reduction and formation of bigger Ag NPs. The realized antibacterial assays showed differences between antimicrobial action of AgBr and Ag nanoparticles and Ag^+^ ions used as reference material. The differences were connected to the interaction of the silver based materials with the cell walls of microorganisms. It was clearly proved that from the studied silver materials the gram-positive bacteria strains are most sensitive to Ag NPs probably due to destructive interaction of these NPs with the cell walls. On the other hand, AgBr NPs are more efficient against gram-negative bacteria and yeast microbial strains due to the combination of interaction of solid AgBr nanoparticles with cell walls and simultaneous penetration of Ag^+^ ions into the cells. This observation of different antimicrobial activity of various forms of NPs based on the metallic silver or silver bromide respectively could be the guideline for the future formulations of the antimicrobials targeted to have a specific mode of action against different types of microbial strains.

## Supporting Information

S1 Fig Effective diameter of AgBr nanoparticles prepared in the presence of used polymers obtained by DLS technique. (TIF)

S2 Fig Effective diameter of Ag nanoparticles prepared in the presence of used polymers obtained by DLS technique. (TIF)

S3 Fig Cyclic voltammograms of Ag^+^ ions in the presence of PEG, PVP, PVA and HEC under the scanning rate equal to 0.1 V/s. (TIF)

S4 Fig Cyclic voltammograms of Ag^+^ ions in the presence of PEG, PVP, PVA and HEC under the scanning rate equal to 1 V/s. (TIF)

S1 Table The concentrations (mg/L) of Ag^+^ ions in studied systems. (DOCX)
