# Supplementary figures and images for: Comparative Study of Antimicrobial Activity of AgBr and Ag Nanoparticles (NPs)
Source: PLoS One. 2015 Mar 17;10(3):e0119202. doi: 10.1371/journal.pone.0119202 (PMC4363559; doi:10.1371/journal.pone.0119202)

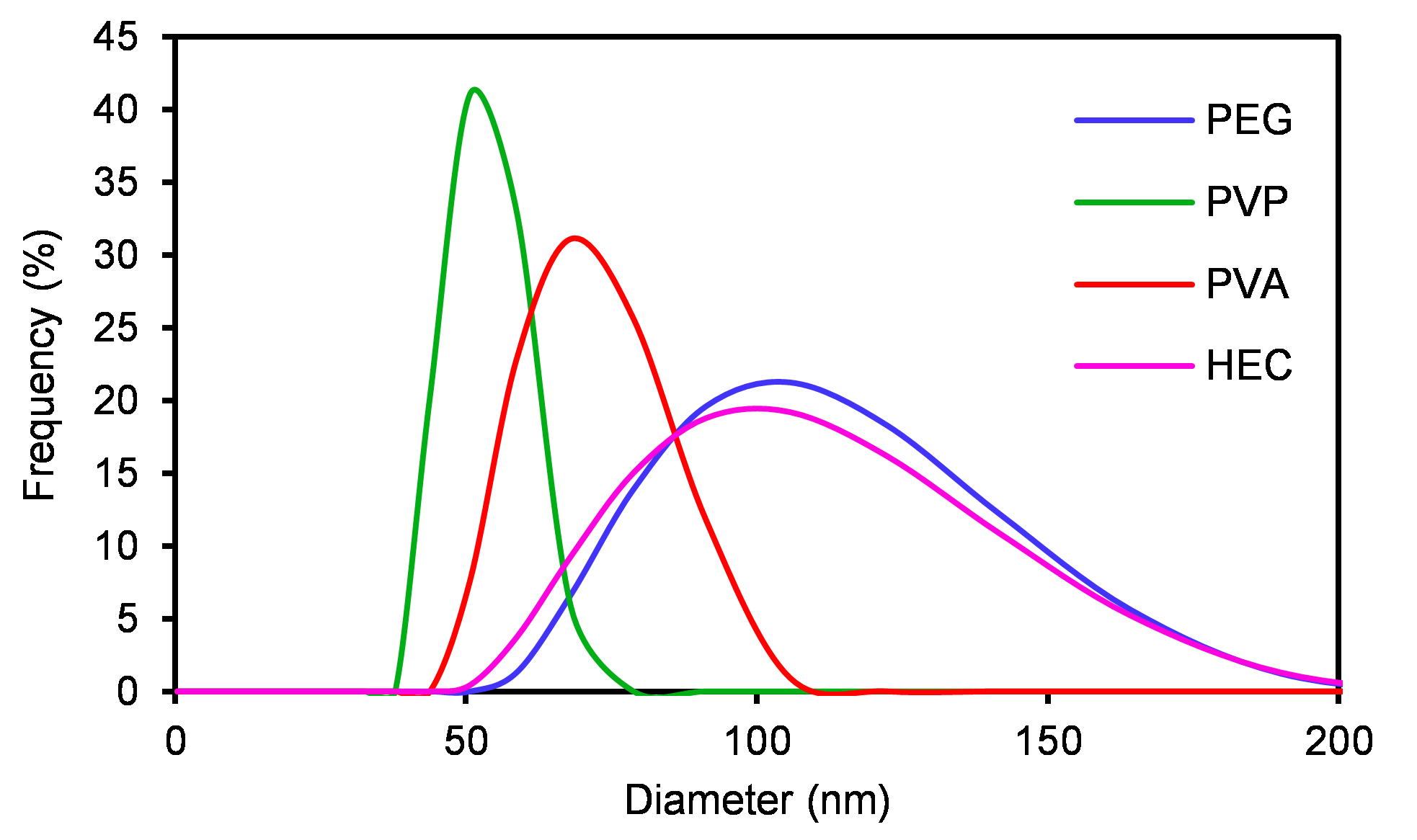

Supplement: S1 Fig — (TIF) [file pone.0119202.s001.tif]

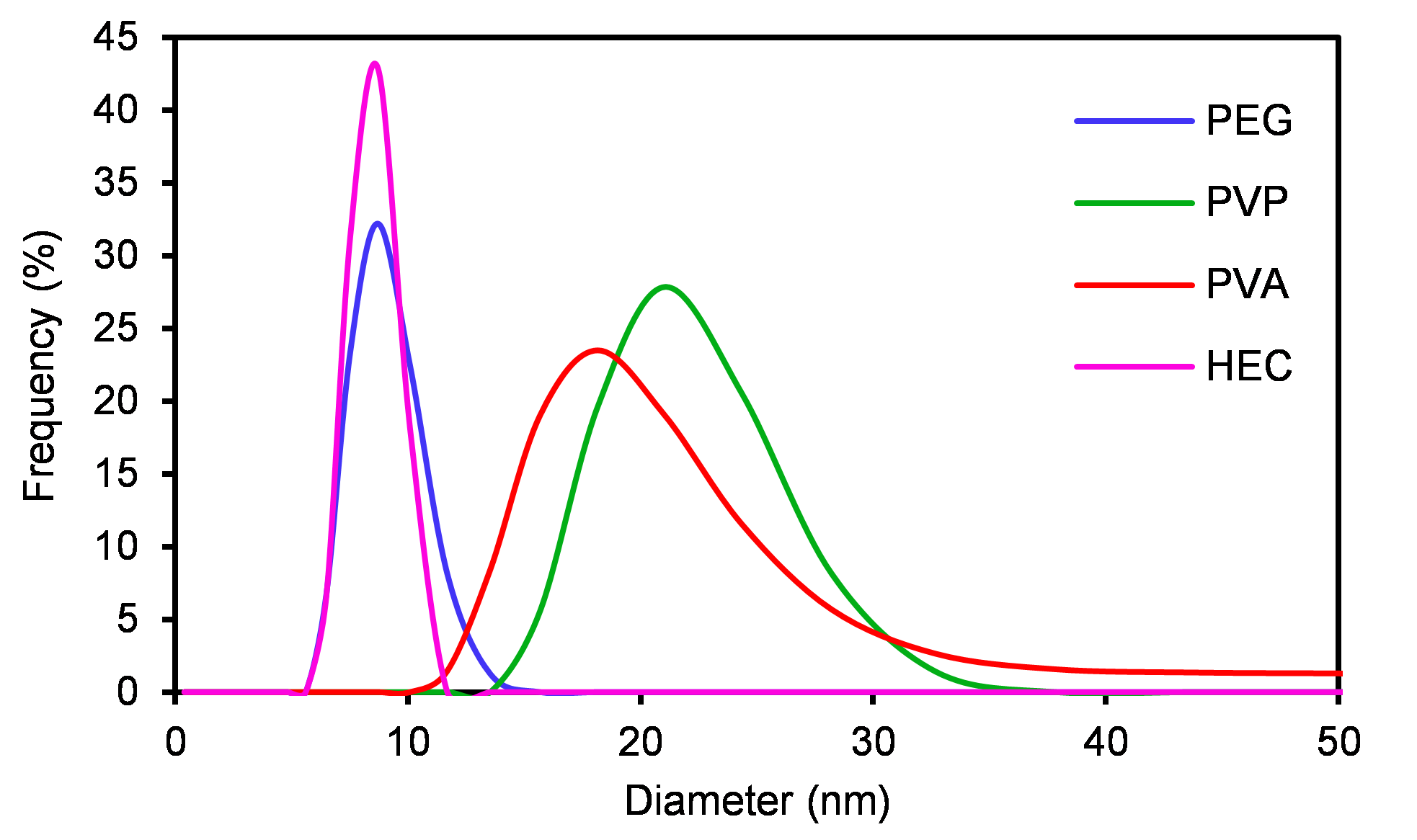

Supplement: S2 Fig — (TIF) [file pone.0119202.s002.tif]

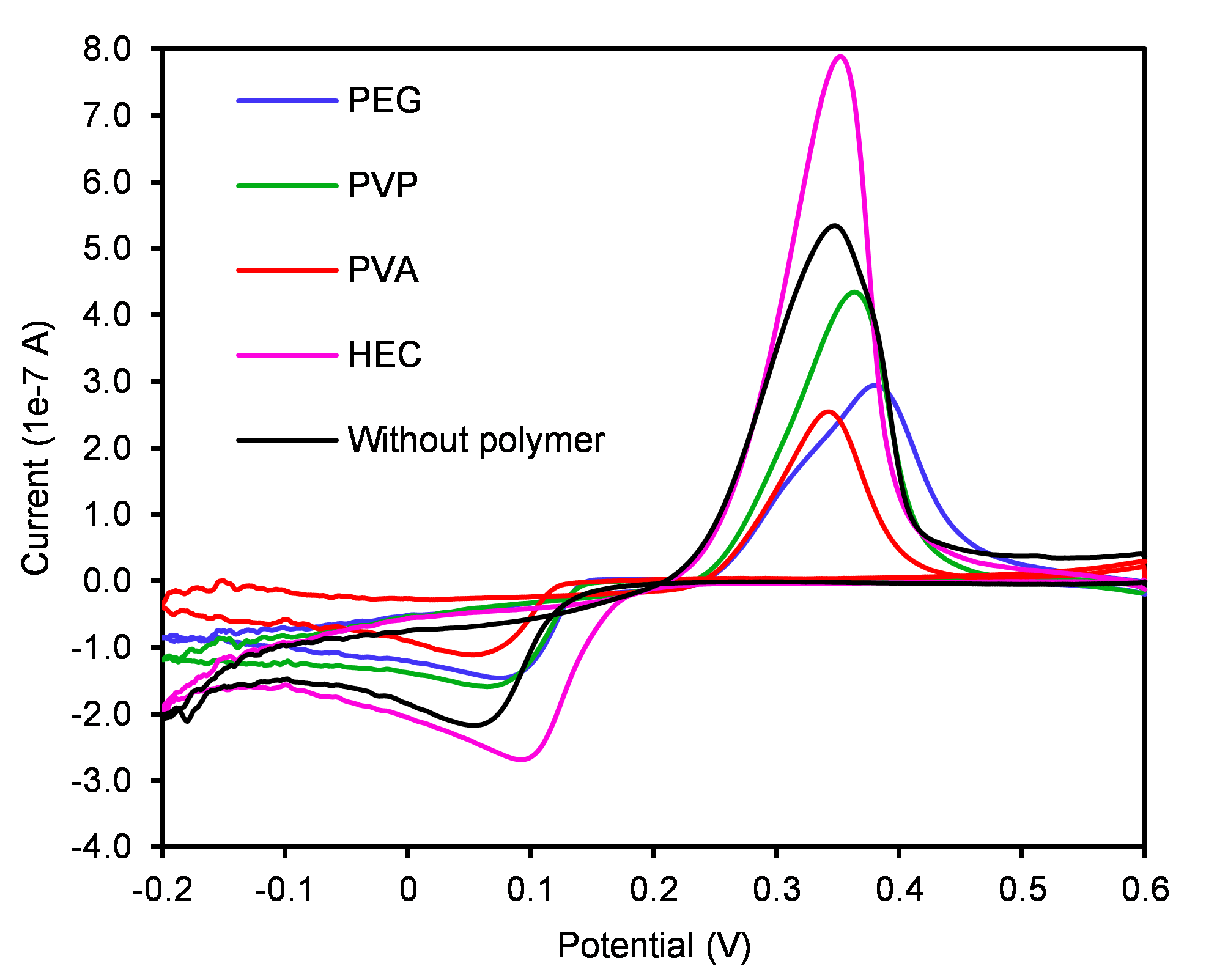

Supplement: S3 Fig — (TIF) [file pone.0119202.s003.tif]

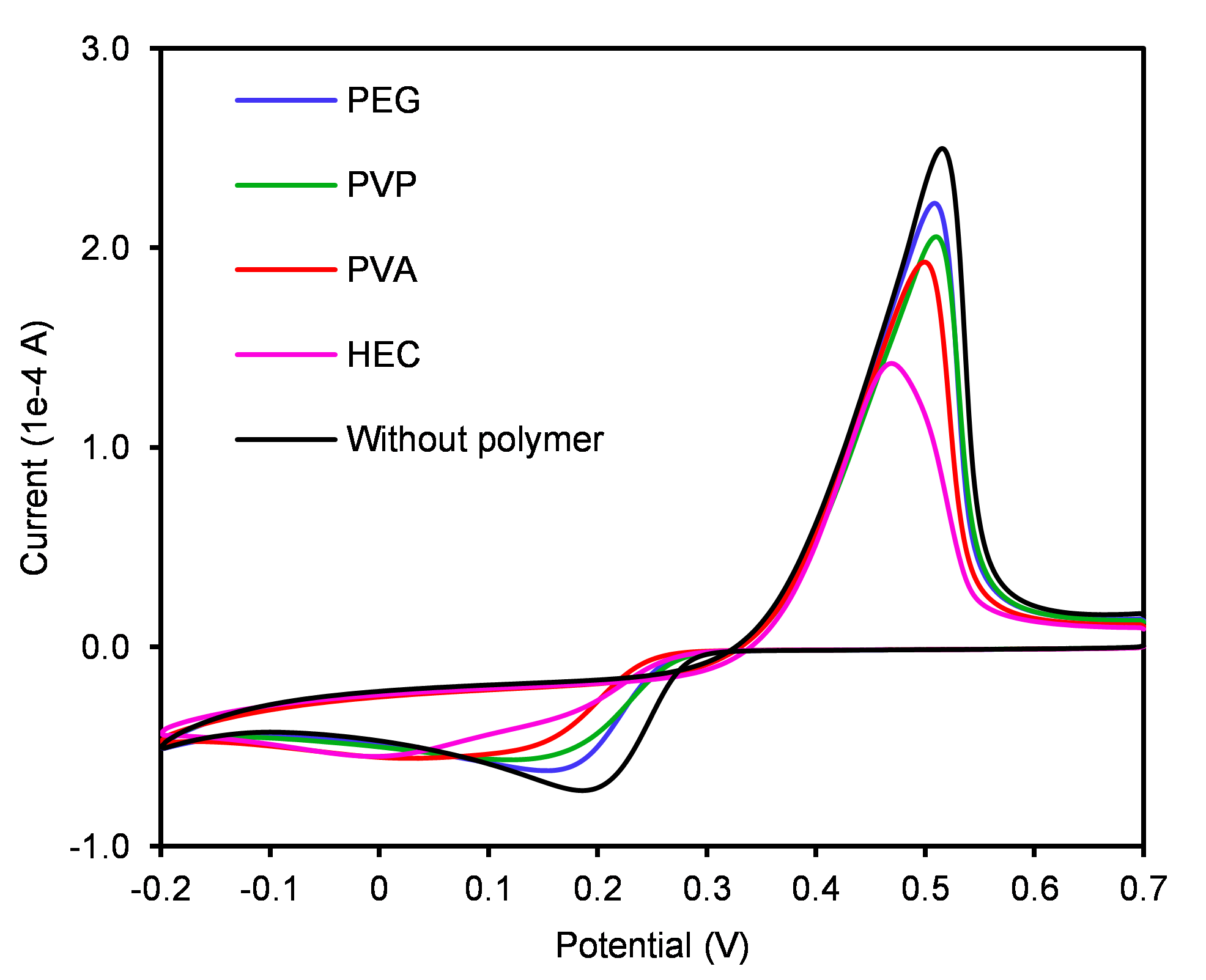

Supplement: S4 Fig — (TIF) [file pone.0119202.s004.tif]
