# Supplementary material for: Comparative Study of Antimicrobial Activity of AgBr and Ag Nanoparticles (NPs)
Source: PLoS One. 2015 Mar 17;10(3):e0119202. doi: 10.1371/journal.pone.0119202 (PMC4363559; doi:10.1371/journal.pone.0119202)
Supplement: S1 Table — (DOCX) [file pone.0119202.s005.docx]

Table S1. The concentrations (mg/L) of Ag^+^ ions in studied systems.

|  | AgBr | Ag |
| --- | --- | --- |
| PEG | 4.93·10^-5^ | 7.30·10^-6^ |
| PVP | 2.75·10^-5^ | 1.42·10^-5^ |
| PVA | 4.06·10^-5^ | 1.31·10^-5^ |
| HEC | 7.28·10^-5^ | 2.65·10^-6^ |
